# Effectiveness of a community-based nutritional intervention on Mediterranean Diet adherence in rural older adults: A quasi-experimental study

**DOI:** 10.1016/j.aprim.2026.103575

**Published:** 2026-07-07

**Authors:** Maria Esther Cortes-Fernandez, Eva Cortes-Fernandez, Maria Jose Zarzuelo

**Affiliations:** a Department of Pharmacy and Pharmaceutical Technology, University of Granada, Spain; b Distrito Centro de Almería, Spain

**Keywords:** Mediterranean Diet, Rural health, Primary health care, Community pharmacy services, Health literacy, Quasi-experimental study, Dieta Mediterránea, Salud rural, Atención primaria de salud, Servicios de farmacia comunitaria, Alfabetización en salud, Estudio cuasi-experimental

## Abstract

**Objective:**

To evaluate the effectiveness of a community intervention on lifestyle in adherence to the Mediterranean Diet and the metabolic health of a highly vulnerable rural population.

**Design:**

12-Month prospective quasi-experimental study.

**Setting:**

Sparsely populated rural primary care areas in the south of Spain.

**Participants:**

187 adults aged ≥ 65 years, allocated to a Control Group (CG: *n* = 96) and an Intervention Group (IG: *n* = 91). 91.4% reported low socioeconomic status and 85% possessed only primary education or were illiterate.

**Interventions:**

The IG received personalized nutritional counseling and educational workshops led by community pharmacists, designed to complement standard primary care. The CG received standard care.

**Main measurements:**

The primary outcome was Mediterranean Diet adherence (MEDAS-14 test). Secondary outcomes included physical activity levels, body mass index, waist circumference, and lipid profiles (total cholesterol, triglycerides, LDL, HDL).

**Results:**

Following the 12-month intervention, the IG exhibited a profound behavioral shift: the proportion of participants with high Mediterranean Diet adherence skyrocketed from 51.6% to 92.3%, and those engaging in high physical activity increased almost sixfold (6.6–38.5%). The CG showed no lifestyle improvements. The IG achieved progressive, statistically significant reductions in waist circumference, triglycerides, and LDL-cholesterol (Rosenthal's *r* ≈ 0.50).

**Conclusions:**

A pharmacist-led intervention effectively overcomes severe health literacy barriers. This task-shifting model induces massive lifestyle changes and mitigates cardiometabolic risk, relieving overburdened rural primary care teams.

## Abstract (es)

**Objetivo:**

Evaluar la efectividad de una intervención comunitaria sobre el estilo de vida en la adherencia a la Dieta Mediterránea y la salud metabólica de una población rural altamente vulnerable.

**Diseño:**

Estudio prospectivo cuasi-experimental de 12 meses de duración.

**Emplazamiento:**

Zonas rurales despobladas de atención primaria en el sur de España.

**Participantes:**

187 adultos ≥65 años, asignados a un Grupo Control (GC: n = 96) y un Grupo Intervención (GI: n = 91). El 91,4% reportó bajo nivel socioeconómico y el 85% tenía solo estudios primarios o era analfabeto.

**Intervenciones:**

El GI recibió consejo nutricional personalizado y talleres educativos adaptados, liderados por farmacéuticos comunitarios como apoyo a la atención primaria. El GC recibió atención habitual.

**Mediciones principales:**

El resultado principal fue la adherencia a la Dieta Mediterránea (cuestionario MEDAS-14). Los resultados secundarios incluyeron niveles de actividad física, índice de masa corporal, perímetro abdominal y perfil lipídico.

**Resultados:**

Tras 12 meses, el GI mostró un profundo cambio conductual: la proporción con alta adherencia a la Dieta Mediterránea aumentó del 51,6% al 92,3%, y aquellos con alta actividad física casi se multiplicaron por seis (del 6,6% al 38,5%). El GC no mostró mejoras. El GI logró reducciones progresivas y estadísticamente significativas en el perímetro abdominal, triglicéridos y colesterol LDL (r de Rosenthal ≈ 0,50).

**Conclusiones:**

La intervención farmacéutica supera eficazmente las barreras de alfabetización en salud. Este modelo de delegación de tareas (“task-shifting”) induce cambios masivos en el estilo de vida y mitiga el riesgo cardiometabólico, aliviando la sobrecarga de los equipos de atención primaria rural.

## Introduction

The Mediterranean Diet is widely recognized as the gold standard for metabolic health and chronic disease prevention.^1^ However, Southern Europe is currently experiencing a “nutritional transition,” where adherence to this traditional dietary pattern is rapidly declining. This phenomenon is particularly concerning in rural populations. Once assumed to be guardians of traditional dietary habits, rural communities are now facing an increasingly obesogenic environment characterized by sedentary lifestyles and the high consumption of ultra-processed foods.^2^, ^3^

In Spain, the severe depopulation of rural areas has exacerbated health inequalities.^4^ Older adults in these municipalities face significant structural barriers to accessing specialized nutritional care, as primary healthcare centers are often understaffed, distant, or lack registered dietitians. Consequently, obesity rates in the rural elderly have risen alarmingly, increasing the burden of metabolic syndrome and cardiovascular disease.^5^

To address this public health gap, community-based strategies and “task-shifting” models are urgently required. Utilizing highly accessible local health agents, such as community pharmacists, offers a viable channel to deliver continuous nutritional education.^6^, ^7^ While previous studies have evaluated pharmacist-led interventions, evidence regarding their impact on comprehensive dietary behavioral change in isolated rural elderly populations remains scarce. The Mediterranean Diet is traditionally characterized by a high intake of plant-based foods, whole grains, and olive oil as the primary fat source, alongside a moderate consumption of fish and low intake of red meat.^8^

In rural primary care settings, General Practitioners and nurses are increasingly overburdened, leaving limited time for the intensive, continuous nutritional counseling required to achieve meaningful behavioral changes in older adults. Consequently, there is an urgent need to implement and evaluate “task-shifting” models that integrate other accessible community health professionals. Community pharmacists, due to their wide geographical distribution in rural “healthcare deserts” and their close relationship with patients, represent an untapped resource to support primary care teams. Therefore, the rationale for this study is to provide robust evidence on whether a structured, pharmacist-led lifestyle intervention can effectively bridge this gap. The aim was to evaluate the effectiveness of this community-based intervention on adherence to the Mediterranean Diet, physical activity, and metabolic risk factors in rural older adults over a 12-month period.

## Methods

### Study design

**Figure 1:**
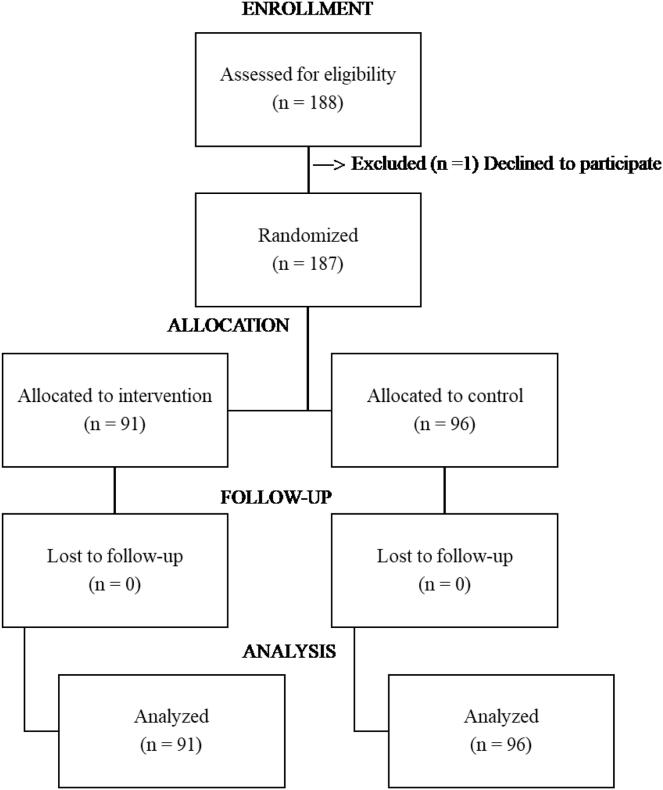
CONSORT flow diagram.

A 12-month, quasi-experimental study was conducted in 51 depopulated rural municipalities (<1000 inhabitants) in a province in southeastern Spain. Participants were allocates in Intervention Group (IG) or in Control Group (CG) (Fig. 1).

The inclusion criteria for the study were: Understanding Spanish, residing in localities with fewer than 500 inhabitants, being aged 65 years or older, and absence of concomitant mental or medical conditions that preclude participation.

The exclusion criteria included were: being under medical treatment with therapeutic prescriptions that require specific diets and consumption of drugs and/or substances that alter perception and/or behavioral autonomy.

The sample size was calculated a priori to detect a clinically relevant difference of 1 point in the primary outcome (MEDAS-14 test) between the intervention and control groups. Assuming a standard deviation of 2.3 points, a statistical power of 80%, and an alpha error of 5% (95% confidence level), a minimum of 84 participants per group were required. Anticipating a 10% dropout rate, the target recruitment was set at 94 participants per group (188 in total). Ultimately, 187 participants from the rural municipalities of Almería were successfully recruited and allocated (Fig. 1).

### Intervention

The intervention was designed to complement standard primary care. The CG received standard care, which consisted of basic, non-individualized written health advice regarding general nutrition.

The IG participated in a structured lifestyle modification program led by trained community pharmacists. The protocol consisted of:

- Personalized Counseling: Face-to-face individual visits at baseline, 1, 3, 6, and 12 months. During these 30-min sessions, pharmacists set individualized goals based on the PREDIMED guidelines study (e.g., substituting sunflower oil with extra virgin olive oil, increasing tree nut consumption to ≥3 times/week, and replacing red meat with poultry or fish).
- Educational Workshops: Quarterly group sessions (45 min) designed for populations with low health literacy. Topics included practical label reading, economic healthy cooking, and strategies to increase daily movement.

The study protocol was approved by the Research Ethics Committee of the province of Almeria (approval code: 10012025) in January 2023. Written informed consent was obtained from all participating patients.

### Data collection and assessment tools

Data were collected by the researchers at baseline and at all follow-up visits.

• Dietary Adherence: Evaluated using the validated 14-item MEDAS screener questionnaire, administered via face-to-face interview to prevent reading comprehension barriers. Scores range from 0 to 14. A score of ≥9 points was categorized as “High Adherence”, while <9 was considered low/medium adherence.^9^
• Physical Activity Assessment (IPAQ). Physical activity was self-reported and categorized based on standard criteria (e.g., adapted International Physical Activity Questionnaire) into three levels: Low (sedentary, <150 min/week of moderate activity), Moderate (150–300 min/week), and High (>300 min/week or vigorous activity).^10^
• Anthropometric and Biochemical Parameters: Body Mass Index (BMI) and waist circumference were measured using standardized ISAK protocols. Fasting blood samples were analyzed for total cholesterol, HDL, LDL, triglycerides, and glycated hemoglobin (HbA1c) at the local reference laboratories.
• Meal routines were assessed using the following questions: Do you eat at home? Do you eat alone or with others? Do you buy your own food? and Do you cook your own food?

### Statistical

Data analysis was conducted using robust statistical method evaluated between-group differences and within-group longitudinal changes over the 12-month follow-up.

Categorical variables (such as gender, educational level, and adherence categories) were expressed as frequencies and percentages, and comparisons were performed using Pearson's Chi-square test. Cramer's *V* was calculated to determine the effect size of categorical associations. For continuous variables, data were expressed as mean ± standard deviation (SD). Normality was assessed prior to analysis.

To evaluate baseline homogeneity and cross-sectional differences between the Intervention and Control Groups, the Mann–Whitney *U* test was employed for non-parametric continuous variables. Within-group changes from baseline to month 12 were evaluated using the Wilcoxon signed-rank test, reporting Rosenthal's *r* statistic to quantify the clinical effect size (categorized as small: ∼0.10, medium: ∼0.30, large: ≥0.50).

Finally, to comprehensively assess the longitudinal impact of the intervention across all five time points (Baseline, 1, 3, 6, and 12 months), a Repeated Measures Analysis of Variance (ANOVA) was conducted. The ‘Time × Group’ interaction was the primary indicator of the intervention's specific effect. When the assumption of sphericity was violated (assessed via Mauchly's test), Greenhouse–Geisser or Huynh–Feldt corrections were applied. Effect sizes for the longitudinal models were reported as partial eta-squared (*ηp*^2^). All analyses were performed on an Intention-to-Treat (ITT) basis, adjusting for baseline imbalances, with statistical significance established at a two-sided *p*-value < 0.05.

## Results

A total of 187 participants completed the baseline assessment and were allocated to the CG (*n* = 96) and the IG (*n* = 91). The sample had a mean age of 73.63 ± 5.53 years, with a slight predominance of females (51.9%) over males (48.1%). Socioeconomic analysis reflected the context of the depopulated rural areas studied, where an overwhelming majority of participants (91.4%) reported a low economic level, and 84.5% had only primary education or were illiterate.

At baseline, the groups were homogeneous regarding parameters such as BMI, waist circumference, HDL-cholesterol levels, and HbA1c, with no statistically significant differences. However, initial differences were observed in triglyceride levels (statistically higher in the CG) and LDL-cholesterol (statistically higher in the IG) (*p* < 0.001). These baseline differences were adjusted for in the subsequent longitudinal statistical models (Table 1).

**Table 1:** Sociodemographic data.

	CG		IG		Total	
	*n*	%	*n*	%	*N*	%
Gender (female)	41	42.7	56	61.5	97	51.9
Economical level (low)	87	90.6	84	92.3	171	91.4
Educational level (primary)	70	72.9	73	80.2	143	76.5
Eat alone (yes)	47	49.0	42	46.2	89	47.6
Physical activity (medium)	54	56.3	41	45.1	96	51.3

*Mediterranean Diet adherence*						
Low	3	3.1	1	1.1	4	2.1
Medium	77	80.2	43	47.3	120	64.2
High	16	16.7	47	51.6	63	33.7

	Mean	SD	Mean	SD	Mean	SD
Age (years)	72.4	5.3	74.9	5.5	73.6	5.5

SD: standard deviation. CG: Control Group. IG: Intervention Group.

### Impact on Mediterranean Diet adherence and physical activity

The structured intervention led to a progressive and marked improvement in lifestyle habits. Analysis of the dietary dimension revealed that longitudinal tracking of frequencies showed a massive behavioral shift in the IG. By month 12, the proportion of IG participants reaching “High Adherence” to the Mediterranean Diet skyrocketed from 51.6% to 92.3%. In stark contrast, the CG showed a slight deterioration, with high adherence dropping from 16.7% to 14.6% (Table 2).

**Table 2:** Changes in Mediterranean Diet adherence and physical activity levels from baseline to month 12.

Lifestyle variable	Category	Control group baseline (*n* = 96)	Control group month 12 (*n* = 96)	Intervention group baseline (*n* = 91)	Intervention group month 12 (*n* = 91)
Mediterranean Diet Adherence (MEDAS-14)	Low	3 (3.1%)	3 (3.1%)	1 (1.1%)	0 (0.0%)
	Medium	77 (80.2%)	79 (82.3%)	43 (47.3%)	7 (7.7%)
	High (≥9 points)	16 (16.7%)	14 (14.6%)	47 (51.6%)	84 (92.3%)

Physical activity level	Low	30 (31.3%)	30 (31.3%)	44 (48.4%)	9 (9.9%)
	Moderate	54 (56.3%)	54 (56.3%)	41 (45.1%)	47 (51.6%)
	High	12 (12.5%)	12 (12.5%)	6 (6.6%)	35 (38.5%)

Similarly, physical activity levels improved remarkably over the 12 months solely in the intervention arm. The percentage of IG individuals engaged in “High” physical activity multiplied almost sixfold, rising from 6.6% at baseline to 38.5% at month 12. Meanwhile, those reporting “Low” physical activity dropped drastically from 48.4% to just 9.9%. Strikingly, the physical activity distribution in the CG remained entirely static throughout the entire year, further proving the efficacy of the active pharmacist-led counseling (Table 2).

### Evolution of anthropometric and metabolic parameters

The comprehensive monitoring across five time points (Baseline, month 1, 3, 6, and 12) allowed for a robust evaluation of the intervention's clinical impact (Table 3).

**Table 3:** Temporal evolution of anthropometric and metabolic parameters by study group (Control vs. Intervention).

Parameter	Group	Baseline	Month 1	Month 3	Month 6	Month 12
BMI (kg/m^2^)	CG	30.53 ± 1.81	30.51 ± 1.81	30.52 ± 1.81	30.54 ± 1.81	30.55 ± 1.82
	IG	29.96 ± 2.62	29.87 ± 2.61	29.71 ± 2.57	29.46 ± 2.57	29.18 ± 2.52

Waist circumference (cm)	CG	101.53 ± 7.65	101.19 ± 8.55	101.47 ± 7.67	101.53 ± 7.67	101.62 ± 7.70
	IG	103.20 ± 6.82	102.87 ± 6.75	102.03 ± 6.61	101.01 ± 6.52	99.77 ± 6.56

Total cholesterol (mg/dL)	CG	174.99 ± 41.72	173.29 ± 39.35	172.51 ± 39.23	172.74 ± 38.16	172.90 ± 38.50
	IG	181.82 ± 28.87	176.30 ± 28.30	173.12 ± 24.99	169.12 ± 23.67	164.80 ± 22.50

Triglycerides (mg/dL)	CG	129.18 ± 32.71	128.86 ± 32.23	128.69 ± 31.57	129.28 ± 32.41	129.40 ± 32.50
	IG	115.42 ± 23.98	112.79 ± 22.66	111.92 ± 22.80	110.33 ± 20.95	107.80 ± 20.10

LDL-cholesterol (mg/dL)	CG	109.34 ± 18.62	108.76 ± 16.92	108.26 ± 16.22	108.98 ± 16.14	109.10 ± 16.50
	IG	121.66 ± 23.35	118.54 ± 22.96	117.16 ± 21.79	115.60 ± 21.72	112.05 ± 21.10

HDL-cholesterol (mg/dL)	CG	49.78 ± 7.81	49.90 ± 7.80	49.85 ± 7.75	49.80 ± 7.70	49.75 ± 7.65
	IG	50.15 ± 5.45	51.90 ± 5.30	52.70 ± 5.40	53.90 ± 5.50	55.10 ± 5.60

HbA1c (%)	CG	6.01 ± 0.16	6.01 ± 0.16	6.02 ± 0.16	6.02 ± 0.16	6.02 ± 0.17
	IG	5.99 ± 0.10	5.98 ± 0.10	5.95 ± 0.10	5.89 ± 0.11	5.82 ± 0.12

The IG achieved a progressive, statistically significant reduction in both BMI and Waist Circumference. Wilcoxon signed-rank tests for paired samples within the IG confirmed that the anthropometric improvements were significant at every follow-up phase. Notably, the reduction in waist circumference at 12 months yielded a strong clinical effect size (*Z* = −6.70, *p* < 0.001, Rosenthal's *r* = −0.49), mobilizing visceral fat effectively. In stark contrast, the CG showed no significant anthropometric changes, with waist circumference stabilizing around 101.6 cm by the end of the year.

Regarding the metabolic profile, the nutritional counseling triggered immediate and sustained biochemical benefits in the IG. From baseline to month 12, the IG experienced drastic, statistically significant reductions in total cholesterol (*Z* = −6.94, *p* < 0.001, *r* = −0.51), triglycerides (*Z* = −5.29, *p* < 0.001, *r* = −0.39), and LDL-cholesterol (*Z* = −6.76, *p* < 0.001, *r* = −0.50). Concurrently, HbA1c levels in the IG showed a favorable descending trend (from 5.99% to 5.82%), effectively halting the glycemic progression observed in the CG (Table 3).

To address potential sex-specific variations in the response to the community-based nutritional intervention, a comparative analysis was performed between male and female participants across the 12-month follow-up period (Table 4). Overall, women demonstrated a more rapid and pronounced improvement in several key metabolic and anthropometric parameters compared to men.

**Table 4:** Differences between variables according to the gender.

Variables	Mean (SD) 1 month	Mean (SD) 6 months	Mean (SD) 12 months
*Total cholesterol (mg/dL)*			
Males	−2.57 (5.68)*	−2.30 (6.88)	−3.48 (9.07)*
Females	−4.27 (5.13)*	−4.86 (10.71)	−8.20 (14.67)*

*Triglycerides (mg/dL)*			
Males	−0.65(3.43)*	−0.65(3.85)	−1.85(4.72)
Females	−2.11 (3.67)*	−0.94 (4.64)	−2.33 (6.22)

*HDL (mg/dL)*			
Males	0.96 (1.93)	0.94 (2.12)	1.33 (2.89)
Females	1.08 (1.78)	0.89 (2.43)	1.81 (3.40)

*LDL (mg/dL)*			
Males	−0.59 (4.57)**	−1.09 (3.69)	−2.32 (5.77)
Females	−2.79 (3.95)**	−1.55 (4.46)	−3.76 (7.23)

*HbA1c (%)*			
Males	0.00 (0.04)*	−0.03 (0.10)**	−0.05 (0.11)**
Females	−0.01 (0.03)*	−0.08 (0.11)**	−0.10 (0.13)**

*BMI (kg/m*^*2*^*)*			
Males	−0.04 (0.13)**	−0.14 (0.34)	−0.25 (0.52)
Females	−0.07 (0.07)**	−0.22 (0.50)	−0.37 (0.65)

*Waist circumference (cm)*			
Males	−0.10 (0.34)*	−0.73 (1.23)	−1.08 (1.91)
Females	−0.24 (0.50)*	−1.04 (1.62)	−1.82 (2.53)

*M*: mean, SD: standard deviation, *U*, Mann–Whitney.

* *p* < 0.05.

** *p* < 0.001. BMI: body mass index.

Regarding lipid profiles, women achieved significantly greater reductions in total cholesterol at month 1 (4.27 ± 5.13 mg/dL, *p* < 0.05) and month 12 (8.20 ± 14.67 mg/dL, *p* < 0.05) compared to men. Similarly, women exhibited significantly higher early reductions at month 1 in triglycerides (2.11 ± 3.67 mg/dL, *p* < 0.05) and LDL cholesterol (2.79 ± 3.95 mg/dL, *p* < 0.001), while men showed minor downward trends that did not reach statistical significance between groups.

Furthermore, glycemic control also revealed significant disparities favoring the female subgroup; the decrease in HbA1c levels was markedly more pronounced in women at month 6 (0.08 ± 0.11%, *p* < 0.001) and month 12 (0.10 ± 0.13%, *p* < 0.001) than in men. Finally, anthropometric changes indicated a faster initial response in women, who presented significantly greater decreases at month 1 in both BMI (0.07 ± 0.07 kg/m^2^, *p* < 0.001) and waist circumference (0.24 ± 0.50 cm, *p* < 0.05) than their male counterparts. These outcomes suggest that while the intervention yielded general benefits, the clinical and metabolic response was accelerated and more pronounced in women (Table 4).

## Discussion

This quasi-experimental study shows that a pharmacist-led, community-based intervention triggers profound, sustained lifestyle modifications in an aging, rural population, addressing healthcare barriers in depopulated areas. The 12-month follow-up demonstrates that the intervention dramatically increased Mediterranean Diet adherence—high adherence skyrocketing from 51.6% to 92.3% in the IG—and quadrupled high physical activity rates.^11^, ^12^, ^13^ These behavioral shifts translated into clinically relevant improvements in central adiposity and atherogenic lipids, while the CG faced metabolic stagnation.

A cornerstone finding is the demographic context. Over 91% of participants had a low socioeconomic status, and 85% had only primary education or were illiterate. Health literacy typically barriers dietary interventions, limiting success to highly educated urban cohorts. The success in these vulnerable demographic underscores the value of community pharmacists. Their physical proximity, established trust, and adapted educational workshops successfully bridged the health literacy gap, proving that obesogenic environments in “healthcare deserts” can be modified regardless of educational background.^14^, ^15^

Unlike public health trials reporting mere statistical significance, our analysis confirms large clinical effect sizes. In geriatric populations, massive weight loss is unrealistic and undesirable due to sarcopenia risks. However, the IG achieved targeted reductions in waist circumference (a visceral fat surrogate) alongside dramatic drops in triglycerides and LDL-cholesterol. This mobilization of central adiposity—the primary driver of insulin resistance—highlights the power of combining the Mediterranean Diet with daily movement, rather than focusing on restrictive caloric counting.^2^, ^16^, ^17^

The parallel improvement in physical activity within the IG is critical. While dietary workshops improved nutritional intake, holistic counseling mobilized a traditionally sedentary demographic. Static physical activity levels in the CG confirm that passive health advice is ineffective. The synergistic effect of replacing saturated fats with high-quality nutrients (olive oil, nuts) while increasing energy expenditure likely explains the rapid, sustained lipid profile improvements from the first month.^1^

A relevant finding is the sex-based difference in response velocity and magnitude, with accelerated benefits in females. This phenomenon stems from socio-cultural determinants in rural communities. Women in this age cohort traditionally purchase and prepare household food. This central role grants them greater autonomy and high cooking self-efficacy to immediately implement nutritional recommendations. Conversely, men of these generations depend more on the domestic environment and exhibit a gradual shift in habits.^18^

This results align with literature indicating that women generally show higher adherence to healthy diets and prefer sustainable, plant-based profiles (vegetables, fruits, legumes), reducing processed meats and saturated fats more easily than men.^19^ This optimized transition explains why biochemical parameters like LDL cholesterol and HbA1c decreased more pronouncedly in women during early phases (months 1 and 6).

The study's strengths include its robust design, excellent retention rate, and rigorous longitudinal tracking over five time points, proving behavioral change sustainability. Several limitations exist. First, potential modifications in ongoing cardiovascular medications prescribed by primary care physicians were not strictly monitored, so concurrent adjustments cannot be ruled out. Second, the IG may be subject to the Hawthorne effect, as frequent monitoring by pharmacists could independently drive motivation. Third, biochemical determinations were integrated into routine clinical practice rather than a single batch analysis, introducing potential inter-laboratory variability. Finally, self-reported questionnaires for diet and physical activity are subject to social desirability bias, though mitigated by objective biochemical and anthropometric improvements.

## Conclusion

This study proves that a structured, pharmacist-led intervention effectively improves dietary habits, physical activity, and cardiometabolic risk among vulnerable rural elderly, despite low socioeconomic or educational levels. The resulting clinical benefits validate primary care “task-shifting,” showing that integrating community pharmacists offers a scalable, equitable solution to combat obesity in underserved, depopulated areas.

**What is known on this topic**

• The Mediterranean Diet is widely recognized as the gold standard for metabolic health, but adherence is declining in Southern Europe due to a transition toward obesogenic and sedentary environments.
• Older adults in isolated rural areas face structural barriers to accessing specialized nutritional care, a situation exacerbated by staffing shortages in primary care centers and the heavy workload of physicians and nurses.

**What this study adds**

• This study demonstrates that a structured 12-month intervention led by community pharmacists achieves a massive behavioral change, drastically increasing high adherence to the Mediterranean Diet and physical activity levels among rural elderly individuals.
• These behavioral shifts translated into significant clinical and metabolic improvements—including progressive reductions in BMI, waist circumference, total cholesterol, triglycerides, and HbA1c—overcoming the barriers of low health literacy in this population.
• The clinical and metabolic response was significantly faster and more pronounced in women than in men, which is linked to sociocultural factors related to their autonomy in managing and preparing household meals.

## Ethical considerations

The study protocol was approved by the Research Ethics Committee of the province of Almeria (approval code: 10012025) in January 2023. Written informed consent was obtained from all participating patients.

## Funding

This research received no specific grant from any funding agency, commercial or not-for-profit sectors.

## Conflict of interest

The authors declare no conflict of interest.
